# A specific role for *PRND* in goat foetal Leydig cells is suggested by prion family gene expression during gonad development in goats and mice

**DOI:** 10.1002/2211-5463.12002

**Published:** 2016-01-14

**Authors:** Aurélie Allais‐Bonnet, Johan Castille, Maëlle Pannetier, Bruno Passet, Maëva Elzaïat, Marjolaine André, Fatemeh Montazer‐Torbati, Katayoun Moazami‐Goudarzi, Jean‐Luc Vilotte, Eric Pailhoux

**Affiliations:** ^1^ Biologie du Développement et ReproductionINRA, UMR 1198Jouy‐en‐JosasFrance; ^2^ ALLICEParisFrance; ^3^ Génétique Animale et Biologie IntégrativeINRA, UMR 1313Jouy‐en‐JosasFrance

**Keywords:** Doppel, foetal Leydig, goat, mouse, prion, Shadoo

## Abstract

Three genes of the prion protein gene family are expressed in gonads. Comparative analyses of their expression patterns in mice and goats revealed constant expression of *PRNP* and *SPRN* in both species and in both male and female gonads, but with a weaker expression of *SPRN*. By contrast, expression of *PRND* was found to be sex‐dimorphic, in agreement with its role in spermatogenesis. More importantly, our study revealed that *PRND* seems to be a key marker of foetal Leydig cells specifically in goats, suggesting a yet unknown role for its encoded protein Doppel during gonadal differentiation in nonrodent mammals.

**Abbreviations**
CNS: central nervous system
d*pc*: days *post coïtum*
Dpl: Doppel
d*pp*: days *post partum*
ECL: enhanced chemiluminescence
GPI: glycophosphatidylinositol
IHC: immuno‐histo‐chemistry
m*pp*: months *post partum*
PBS: phosphate saline buffer
qRT‐PCR: quantitative RT‐PCR
Sho: Shadoo
TSE: transmissible spongiform encephalopathy

The prion gene family is mainly composed of three genes; *Prnp*, encoding the prion protein (PrP)*, Prnd*, encoding Doppel (Dpl), both located on the same genomic locus on mouse chromosome 2 and *Sprn,* encoding Shadoo (Sho), located on mouse chromosome 7. These proteins are glycophosphatidylinositol (GPI)‐anchored glycoproteins and they shared some structural homology one with each other. The N‐terminal regions of PrP and Sho are composed of basic repeat regions and of an hydrophobic domain, whereas the C‐terminal regions of PrP and Dpl contain alpha helices 1. PrP plays a pivotal role in transmissible spongiform encephalopathy (TSE), a fatal neurodegenerative disorder affecting animals and humans 2, 3, 4. PrP is almost ubiquitously expressed with higher amount of expression occurring in the central nervous system (CNS). In mice and rams, PrP was found to be expressed in germ cells 5, 6, 7 but the genetic ablation of its gene in mice 8, 9, cattle 10 and goats 11 does not induce a fertility‐associated phenotype and/or major neuronal disorders 12. Thus, the PrP biological function remains elusive even if various roles have been proposed 13, 14.These observations suggested a biological redundancy between PrP and another PrP‐like protein in mammals.

Sho is expressed in the CNS and both Sho and PrP share neuro‐protective properties 15. Using *Sprn* reporter mice, a recent study describes expression of *Sprn* in the male and female gonads suggesting an involvement of Sho in reproduction 16. The *Sprn* mRNA knockdown in *Prnp* ^0/0^ embryos produces early embryonic lethality, suggesting that Sho and PrP could play a role in early developmental stages 17, 18. However, the *Sprn* ablation (*Sprn* ^0/0^) or the double‐knockout of *Sprn* and *Prnp* (*Sprn* ^0/0^/*Prnp* ^0/0^) in mice resulted in no drastic developmental phenotype 19.

Dpl is mainly expressed in the testis of adult mammals. Its ectopic expression induces some neuro‐degeneration in the CNS only in the absence of PrP 20, 21, 22, 23, suggesting a biological link between Dpl and PrP. *Prnd* ablation in mice (*Prnd* ^0/0^) resulted in male infertility characterized by the sperm's inability to perform the acrosome reaction and by an elevated level of oxidative DNA damage 24, 25. The double‐knockout of *Prnd* and *Prnp* (*Prnd* ^0/0^/*Prnp* ^0/0^) only mimicked the effect of the *Prnd* single inactivation 25. Immunohistochemical studies of Dpl were performed in gonads of various species, such as humans, rodents, boars and bovidae. The cellular localization of Dpl depends on the maturation stage of the gonads, on the studied species and the antibodies 12. For instance, in rodents and sheep, Dpl was only detected in germinal and somatic cells in mature testis, whereas in humans, boars and bovine, DPL seems to be present during most of the developing stages of the germ cells and in the Sertoli cells of foetal and mature gonads 26, 27, 28, 29. In goats and bovine, DPL was detected both in immature testis and in young female follicles 28, 30. Nevertheless, these different observations suggested a role of Dpl in early and/or mature sex differentiation 12.

To get deeper into the potential role of the prion protein gene family during gonad development, we report the comparative expression profiles of the three members of the prion protein gene family and the comparative localizations of their encoded proteins during ovary and testis development in two different species: goats and mice. These data suggest that *Prnd* may exert a yet unknown specific role in goat foetal Leydig cells.

## Materials and methods

### Animals and tissue samples

Procedures for handling goats were conducted in compliance with the guidelines for Care and Use of Agricultural Animals in Agricultural Research and Teaching (authorization no. 78–34). All goat foetuses and young goats were obtained from pregnant females, following hormonal treatment as previously described 31.

For mice, animal experiments were carried out in strict accordance with the recommendations in the guidelines of the Code for methods and Welfare Considerations in Behavioral Research with Animals (Directive 86/609EC). Experiments were approved by the Local ethics committee of Jouy‐en‐Josas on the Ethics of Animals Experiments of the author's institution, INRA (Permit Number RTA06‐091). All transgenic animal manipulations were performed according to the recommendations of the Haut Conseil des Biotechnologies (Permit number 6461). All mouse foetuses and pups were obtained from pregnant FVB/N, FVB/N *Prnp* ^0/0^ [quoted in 17] and *Prnd* ^0/0^ females 24.

Day 0 post coïtum corresponds to the day of mating. The genetic sex of all foetuses was determined by PCR amplification of SRY and ZFY/ZFX genes, on liver genomic DNA 31. For each goat sample, one gonad was frozen in liquid nitrogen for molecular analysis; the other one was fixed for immuno‐histological studies. For mice, samples of a same sex were pooled before molecular analysis. Two or 3 gonads were fixed for immuno‐histological studies at each developmental stage. Table 1 summarized the number of individuals used at each developmental stage in mice and goats.

**Table 1:** Number of animals used at each developmental stage for gene expression studies with real‐time PCR

Species	Stages	Sex	Tissues	Number of individual	Number of independent RT
Mouse	12.5 d*pc*	Female	2 Gonads + Mesonephros	9	2
		Male	2 Gonads + Mesonephros	7	2
	13.5 d*pc*	Female	2 Gonads + Mesonephros	6	2
		Male	2 Gonads + Mesonephros	12	2
	14.5 d*pc*	Female	2 Gonads	4	2
		Male	2 Gonads	6	2
	18.5 d*pc*	Female	2 Gonads	6	2
		Male	2 Gonads	9	2
	5 d*pp*	Female	2 Gonads	11	2
		Male	1 Gonad	4	2
	25 d*pp*	Female	1 Gonad	5	2
		Male	1/2 Gonad	4	2
	50 d*pp*	Female	1 Gonad	3	3
		Male	1/2 Gonad	3	3
	4 m*pp*	Female	1 Gonad	3	3
		Male	1/2 Gonad	3	3
	6 m*pp*	Female	1 Gonad	2	2
		Male	1/2 Gonad	3	3
	6–8 m*pp*	Female	1 Gonad	3	3
		Male	1/2 Gonad	3	3
	10–12 m*pp*	Female	1 Gonad	3	3
		Male	1/2 Gonad	3	3
Goat	30 d*pc*	Female	1 Gonads + Mesonephros	1	2
		Male	1 Gonads + Mesonephros	2	4
	32 d*pc*	Female	1 Gonads + Mesonephros	1	2
		Male	1 Gonads + Mesonephros	2	4
	34 d*pc*	Female	1 Gonads + Mesonephros	2	3
		Male	1 Gonads + Mesonephros	2	4
	36 d*pc*	Female	1 Gonads + Mesonephros	3	3
		Male	1 Gonads + Mesonephros	2	4
	41 d*pc*	Female	1 Gonad	4	2
		Male	1 Gonad	4	4
	45 d*pc*	Female	1 Gonad	9	3
		Male	1 Gonad	4	4
	50 d*pc*	Female	1 Gonad	4	3
		Male	1 Gonad	3	4
	60 d*pc*	Female	1 Gonad	2	3
		Male	1 Gonad	2	4
	70 d*pc*	Female	1 Gonad	2	3
		Male	1 Gonad	2	4
	90 d*pc*	Female	1 Gonad	2	3
		Male	Piece of gonad	2	4
	130 d*pc*	Female	1 Gonad	1	3
		Male	Piece of gonad	1	2
	5 d*pp*	Female	Piece of gonad	1	3
		Male	Piece of gonad	1	3
	1 m*pp*	Female	Piece of gonad	1	3
		Male	Piece of gonad	1	4
	3 m*pp*	Female	Piece of gonad	1	3
		Male	Piece of gonad	1	4
	7 m*pp*	Female	Piece of gonad	1	3
		Male	Piece of gonad	1	4
	4 y*pp*	Male	Piece of gonad	1^a^	3

a Three different pieces of the same testis was used to realize three independent RT.

### PCR primers

PCR primers were designed using primer express Software for Real‐Time PCR 3.0 (Applied Biosystems, ThermoFisher Scientific, Courtaboeuf, France) analysis of *Prnp, Prnd* and *Sprn* expression in mice and goats (Table 2). Mice and goats gene sequences were obtained from GenBank. Primer efficiencies and specificities were evaluated on genomic DNA. The chosen sets of primers share similar efficiencies (not below 90%).

**Table 2:** Primers used in the present study

Gene	Gene ID	Ref seq	Forward primer	Reverse primer	E‐efficiency	Amplicon size (bp)
Mice primers						
*Prnp*	ENSMUSG00000079037	NM_011170	TTTTCTCCTCCCCTCCTGTCA	ACCACGAGAATGCGAAGGAA	1.896	100
*Prnd*	ENSMUSG00000027338	NM_023043	CGGGAGAAGCAGGATAGCAA	CTCCCCTTTCCAGCCAGAA	1.846	100
*Sprn*	ENSMUSG00000045733	NM_183147	GAACCGACCGAGGAGTCTACAG	GGTCTAAGGCCGAAGC	1.89	125
*Hprtl*	ENSMUSG00000025630	NM_013556	AAGACTTGCTCGAGATGTCATGAA	ATCCAGCAGGTCAGCAAAGAA	1.912	100
*Ywhaz*	ENSMUSG00000022285	NM_011740	TGGCAGCCTGCATGAAGTC	CGGGCTCCTACAACGTTTTTAT	1.966	100
*H2afz*	ENSMUSG00000037894	NM_016750	CAGCTGTCCAGTGTTGGTGATT	CTAATTAAGCCTCCAACTTGCTCAA	1.938	100
Goat primers						
*PRNP*	ENSBTAG00000027937	NM_001271626	TGCAGGTAACACAGCCAGCTA	TTCGTATTATGCTCATTCCTTGTGA	1.98	100
*PRND*	ENSBTAG00000011010	NM_174158	AGTTGGCTTGTTCATCATTGCA	CCTGGCACATTCTTTTATCTGCTTA	2.00	100
*SPRN*	ENSBTAG00000047474	NM_001080321	AGGAATGATGGCGGCAAAA	GGAGGCACTTGTCCTGAGTGA	1.97	102
*3βHSD*	ENSBTAG00000006769	NM_174343	GCACCTTGTACACTTGTGCCC	GAT GCCGTTGTTATT CAAGGC	2.10	101
*ACTB*	ENSBTAG00000026199	NM_173979	CAGCAAGCAGGAGTACGATGAG	AAGGGTGTAACGCAGCTAACAGT	1.90	85
*YWHAZ*	ENSBTAG00000000236	NM_174814	GGAGCCCGTAGGTCATCTTG	CTCGAGCCATCTGCTGTTTTT	1.95	85
*H2AFZ*	ENSBTAG00000004428		GCGTATTACCCCTCGTCACTTG	CAGCAATTGTAGCCTTGATGAGA	1.97	80

### Quantitative RT‐PCR

RNAs were extracted using the RNeasy Mini kit (Qiagen, Courtaboeuf, France). Super‐Script II (Invitrogen, ThermoFisher Scientific) was used to synthesize cDNA for qRT‐PCR from 1μg (mice) or 2 μg (goats) of gonad RNA (Table 1). To identify appropriate qRT‐PCR normalizing genes for foetal and postnatal gonads in mice, expression stability of seven genes (*Gapdh, Actb, B2 m, Mapk1, H2afz, Ywhaz* and *Hprt1*) was tested at each time point and the GeNorm program 32 used to select a combination of the most stable genes. The three retained genes were *Ywhaz*,* H2afz* and *Hprt1* (Table 2). For goats, the previously described *ACTB*,* YWhAZ* and *H2AFZ* genes were used 33 (Table 2). qRT‐PCR was performed on all genes at all time points, in triplicates, using the Absolute Blue SYBR Green ROX mix (ThermoFisher Scientific) and the StepOnePlus Real‐Time PCR System (Applied Biosystems). The results were analysed by the relative standard curve method with the qbase Software 34. Data points were plotted using Excel. Statistical analyses were performed using the invivostat software 35 that combines an ANOVA approach followed by a Fisher's Least Significant Difference (LSD)‐test.

### Immunostaining

Freshly dissected gonads were fixed in 4% paraformaldehyde in phosphate saline buffer (PBS) at 4 °C for 1 h or overnight (according to the size of the gonad). After washes in PBS with increasing concentrations of sucrose (0, 12%, 15% and 18%), tissue specimens were embedded in Jung Tissue Freezing Medium (Leica Biosystems, Nanterre, France) and frozen at −80 °C. Cryo‐sections (7 μm thick) were obtained and stored at −80 °C until used. The sections were air‐dried, rehydrated in PBS and permeabilized during 30 min in PBS with 0.5% triton and 1% BSA. The primary antibodies were then applied overnight at 4 °C. Table 3 describes the antibody references and concentrations 15, 28, 36, 37, 38. After several washes, the sections were incubated with secondary antibodies for 1 h at room temperature. The slides were then rinsed in PBS, mounted in Vectashield mounting medium with DAPI (Vector) and observed as above.

**Table 3:** Antibodies used in the present study

Primary antibody	Reference	Source	IHC dilution	WB dilution
DPL – purified boDpl67–81	Gift of Dr Paltrinieri (Rondena *et al*. 28)	Rabbit	1 : 150	
PRP – Sha31	(Feraudet *et al*. 36)	Mouse	1 : 1000	
Biotinylated PRP – b Sha31	(Feraudet *et al*. 36)	Mouse		1 : 50 000
Murine SHO	Gift of Dr Westaway (Watts *et al*. 15)	Rabbit	1 : 500	
Ovine SHO	Gift of Dr Peelman (Lampo *et al*. 37)	Rabbit	1 : 50	
Cyp17A	Gift of Dr Hales (Hales *et al*.^38^)	Rabbit	1 : 200	

Secondary antibody	Reference	Conjugate	IHC dilution	WB dilution
Anti‐rabbit IgG‐Dylight 488	072‐03‐15‐06, KPL	Fluorescein	1 : 200	
Anti‐rabbit IgG‐Dylight 594	072‐09‐15‐06, KPL	Fluorescein	1 : 200	
Anti‐mouse IgG‐Cy3	AP160C, Millipore	Cyanine 3	1 : 200	
Anti‐rabbit IgG‐B	BA‐1000, Vector	Biotin	1 : 200	
Anti‐mouse‐IgG‐HRP strep		Horseradish peroxydase Streptavidin		1/20 000
Fluorescein Streptavidin	SA‐5001, Vector		1 : 200	
Texas Red^®^ Streptavidin	SA‐5006, Vector		1 : 200	
Mounting medium	Reference			
Vectashield for fluorescence with DAPI	H‐1200, Vector			

### Western blot analysis

Frozen tissues of adult mice and goats were homogenized in 50 nm Tris HCl, 150 mm NaCl, 0.5% sodium deoxycholate (w/v), 0.1% sodium dodecyl sulphate (w/v), 1% of a nonionic nondenaturing detergent (NP‐40), one complete EDTA free mini‐protease inhibitor tablet per 10 mL (Roche Diagnostic, Saint‐Egrève, France). Whole extracts (20 μg of total protein) were subject to 4–15% gradient SDS/PAGE and transferred to a poly(vinylidene difluoride) membrane (GE healthcare Life Sciences, Vélizy‐Villacoublay, France). The membrane was probed with anti‐biotinylated‐PrP antibody (bSha 31; Table 3). The secondary antibody used was horseradish peroxydase/streptavidin conjugated antirabbit (Table 3). Immunodetection using the enhanced chemiluminescence (ECL) method (PIERCE) was performed according to the manufacturer's instruction and the images were recorded on an image analysis station (Luminescent Image analyse Las‐1000plus Fujifilm).

## Results and discussion

### By contrast with mice, PRND is highly expressed in goat foetal testis

**Figure 1:**
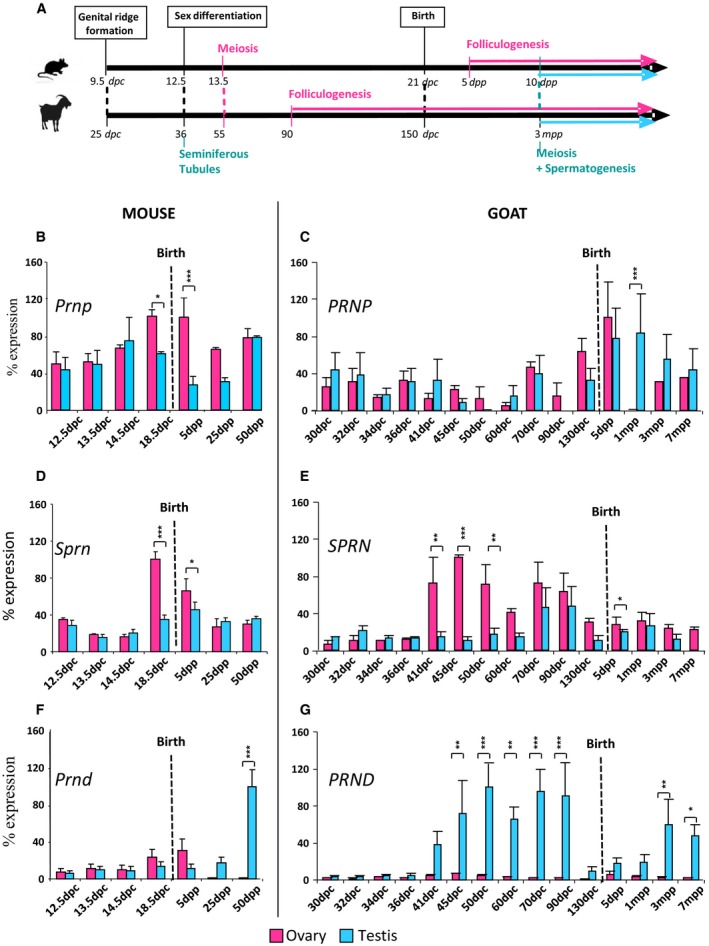
Expression of Prion family genes during gonad development. (A) Chronology of gonad differentiation in mice and goats. Expression of the *Prnp/PRNP* (B, C), *Sprn*/*SPRN* (D, E) and *PrndPRND* (F, G) genes was quantified using real‐time RT‐PCR in ovaries (pink histograms) and testes (blue histograms) in mice (B, D, F) and goats (C, E, G). From 12.5 d*pc* to 13.5 d*pc* (mice) and 30 d*pc* to 36 d*pc* (goats): gonad + mesonephros, others stages: gonad only. *Prnp*/*PRNP*, PrP protein gene; *Sprn/SPRN*, Sho gene; *Prnd*/*PRND*, Dpl gene; d*pc*, days *post coïtum*; d*pp*, days *post partum*; m*pp*, months *post partum*. Values are expressed in percentage according to the highest one noted 100%. Means ± SD were plotted. Planned comparisons were made on the predicted means with ‘sex’ and ‘stage’ as treatment factors (two‐way ANOVA approach, followed by a Fisher's LSD‐test). For each stage, significant differences between the two sexes are showed by stars (*P‐value ≤ 0.05; **P‐value ≤ 0.01; ***P‐value < 0.001).

**Figure 2:**
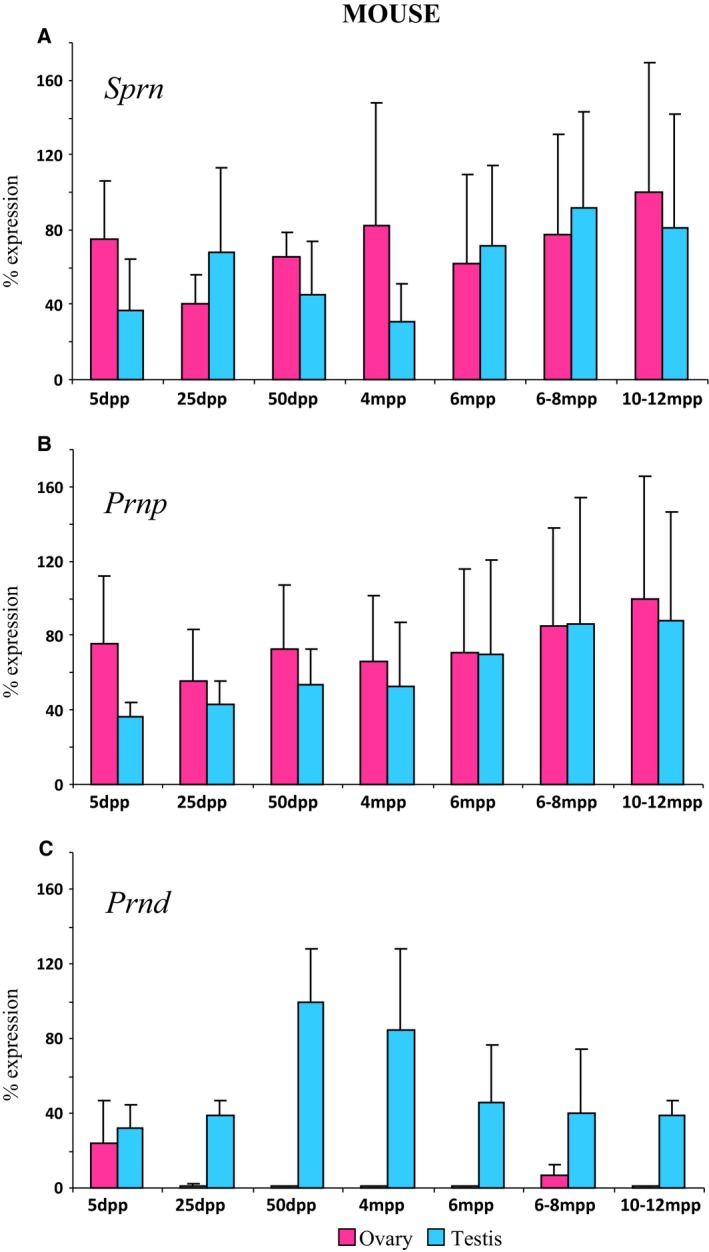
Expression of Prion family genes during postnatal gonad development in mice. *Sprn* (A), *Prnp* (B) and *Prnd* (C) gene expressions were quantified using real‐time RT‐PCR in postnatal ovaries (pink histograms) and testes (blue histograms) in mice. *Sprn*, Sho gene; *Prnp*, PrP protein gene; *Prnd*, Dpl gene; d*pp*, days *post partum*; m*pp*, months *post partum*.

We have previously carried out an expressional study of the Prion gene family in the goat species 30 that suggested an involvement of *PRND* in early gonadal differentiation. The aim of the present study was to complete this observation by (i) including the recently discovered *SPRN* gene and (ii) establishing a comparative view of the expression of the three members of the Prion gene family throughout all gonad developmental stages, from differentiation to adulthood (Fig. 1A), in (iii) two mammalian species, mice and goats. Gonadal expression profiles of *PRNP, SPRN* and *PRND* have been precisely determined using quantitative RT‐PCR instead of classical RT‐PCR as previously used 30, at 7 and 15 developmental stages in mice and goats respectively (Fig. 1B–G), and during ageing in mice where four additional stages were studied. A stable gonadal expression of these three genes in the mouse species was observed during ageing since 50 d*pp* until 10–12 months of age (Fig. 2). In both species, *Prnp/PRNP* gene expression slightly increases during development and appears to be more intense around birth (Fig. 1B,C). During gonadal development, *SPRN* was found to be expressed in male and female gonads of mice and goats at all tested stages (Fig. 1B–E) but only faintly when compared to *PRNP* and *PRND* (as for example in goat testis samples, the cycle thresholds, CT, values are for *PRNP* and *PRND* between 21 and 30, but only between 31 and 36 for *SPRN*). *Sprn/SPRN* appears to be more expressed in ovaries of both species during early follicles formation (i.e. from 18.5 d*pc* to 5 d*pp* in mice and 70–90 d*pc* in goats); and specifically in goat ovaries before the beginning of germ cell meiosis (Fig. 1D,E). Indeed, the highest *SPRN* expression level is found at this premeiotic stage (i.e. 41–50 d*pc*) only in the goat species. The duration of this premeiotic stage, which starts after gonad commitment in one sex or the other (i.e. 12.5 d*pc* in mice, 36 d*pc* in goats), appears quite different in mice and goats, 24 h in mice instead of 2 weeks in goats. Although no profound change could be noticed in mouse ovaries during this period, goat ovaries were organized into cortical and medullar compartments where germ cells were concentrated in the cortex and estrogens were produced by the medulla part under the control of the *FOXL2* gene that has been shown to be a major ovarian‐determining gene in goats, by contrast with mice 39, 40, 41.

Finally and as previously noticed 30, *Prnd/PRND* expression is found higher in testes than in ovaries at adulthood for both species, but this sex‐dimorphism appears since early testicular differentiation specifically in the goat species (Fig. 1F,G). The expression of *Prnd/PRND* in adult testes is well‐documented and *Prnd* has been shown to be required for spermiogenesis in mice 24, 25. However, the concomitant increased level of *PRND* expression with the start of testicular differentiation appears specific to the goat species as it was not observed in mice and it has not been described to date in any other species (Fig. 1G).

### Sho and PrP cellular localizations remain difficult to ascertain

**Figure 3:**
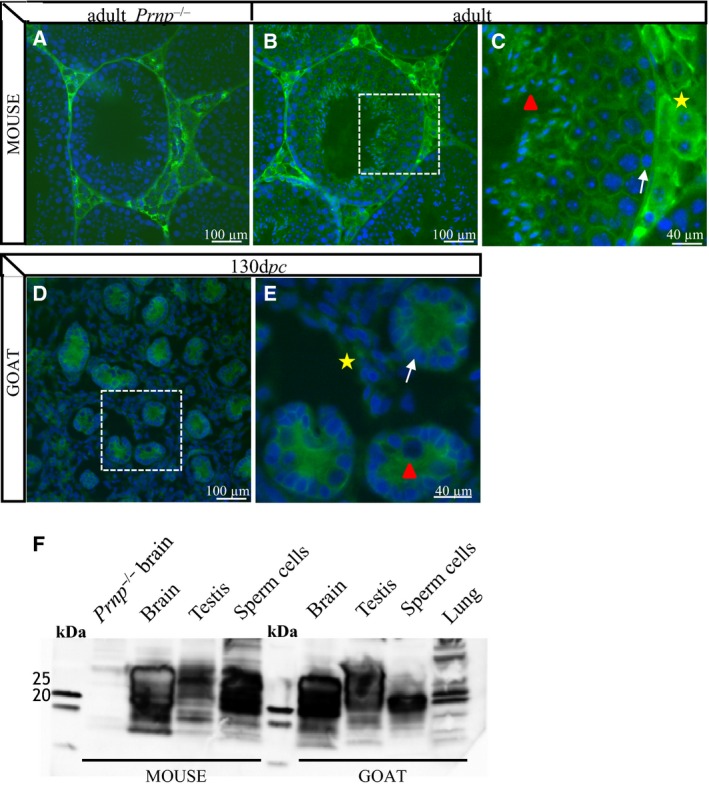
PrP immunodetection in mice and goat testes. (A): The specificity of PrP antibody (sha31) was tested on adult *Prnp* ^0/0^ testis in mice. Testicular interstitial cells showed a nonspecific fluorescence. PrP immunodetection was performed on adult testis in mice (B, C) and on 130 d*pc* testis in goats (D, E). The fluorescent staining is presented with a 4, 6‐diamine‐2‐phenylidole‐dihydrochloride (DAPI) blue nuclear‐specific counterstaining. (C, E) photographs correspond to an enlargement of the white rectangles depicted on (B, D) photographs. Some cells are marked as followed: Sertoli cells (white arrow, pointed on a Sertoli cell nucleus), Leydig cells (yellow star) and germinal cells (red arrowhead). d*pc*, days *post coïtum*. (F): Western blot assays of endogenous PrP (Sha31b) of adult homogenates tissues (brain, testis and lung) and sperm cells in mice and goats. Bands denoted the presence of various PrP isoforms in these tissues. No protein was detected in *Prnp* ^0/0^ brain mice used as negative control.

In order to gain more information on the 3 proteins encoded by the 3 Prion gene family members, we carried out IHC (Immuno‐Histo‐Chemistry) and western blot experiments using available antibodies (Table 3). Two antibodies have been tested against Sho. Each gives a specific staining by IHC that remains different (i) from each other, (ii) from one species to another and also (iii) from Sho gonadal expression already described by additive transgenesis of a *Sprn*‐LacZ mini‐gene in mice 16. Thus, we are unable to ascertain in what gonadal cell type Sho could be detected. The recent derivation of Sho‐knockout mice 19 might help to decipher the real expression pattern of this protein by comparative IHC analysis, but these mice were not available to us at the time of this experiment. In the same way, the PrP protein was detected by IHC and western blot at adulthood in both species and at 130 d*pc* in goats (Fig. 3). By discarding the strong nonspecific staining in the interstitial area of adult mouse testes, identified by using *Prnp* ^0/0^ testicular samples (Fig. 3A), the PrP protein appears to be present at all stages mainly inside the seminiferous tubules, most likely in the Sertoli cells (clearly visible at 130 d*pc* in goats) and in the germ cells at the end of spermiogenesis (see the staining of elongated spermatids on Fig. 3B,C). Presence of PrP in the testis was confirmed by western blot performed on adult mice and goats testes and spermatozoa (Fig. 3F). The presence of PrP on ejaculated sperm cells has already been reported in humans, cattle and mice 5, but its testicular or epididymal origin remains debated, as recently discussed 12. To our knowledge, this is the first time that PrP is clearly detected in the Sertoli cells of immature (130 d*pc* in goats) and mature (in adult mice) testes.

### By contrast with mice, DPL is detected in goat Leydig cells

**Figure 4:**
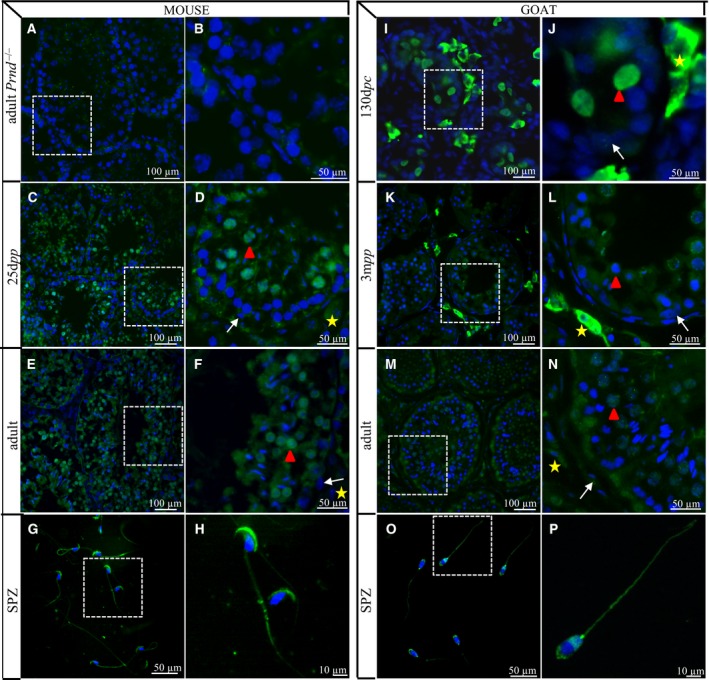
Dpl immunodetection during testis development in mice and goats. In mice, the specificity of the Dpl antibody (boDpl67–81) was tested on adult *Prnd* ^0/0^ testis (A, B). Dpl immunodetection was performed on 25 d*pp* (C, D), adult (E, F) testes and on spermatozoa (G, H). In goats, Dpl immunostaining was carried out 130 d*pc* (I, J), 3 m*pp* (K, L), adult (M, N) testes and on spermatozoa (O, P). The fluorescent staining is presented with a 4, 6‐diamine‐2‐phenylidole‐dihydrochloride (DAPI) blue nuclear‐specific counterstaining. The second (B, D, F, H) and fourth (J, L, N, P) columns correspond to an enlargement of the white rectangles depicted on the first (A, C, E, G) and third (I, K, M, O) columns respectively. Cells are marked as followed: Sertoli (white arrow), Leydig (yellow star) and germinal (red arrowhead). d*pc*, days *post coïtum*; d*pp*, days *post partum*; m*pp*, months *post partum*; SPZ, spermatozoa.

We have previously shown that the DPL protein could be detected in germ cells of both sexes and in foetal Leydig cells of early goats developing testes at 44 and 62 d*pc* 30. According to qRT‐PCR results (Fig. 1F,G) showing a high expression of *PRND* in goat developing testes, we checked its cellular localization throughout testis development in immature and mature testes and on spermatozoa of both species (Fig. 4). First, the specificity of anti‐DPL antibody boDpl67‐81 28 was confirmed as no signal was obtained on adult *Prnd* ^0/0^ mouse testes (Fig. 4A,B). Using this antibody, DPL is detected in the cytoplasm of some germ cells in both species; germ cells that have passed the zygotene stage of meiosis (i.e. pachytene and spermatides)(Fig. 4C,F and K,N) and its presence persists on spermatozoa with a high staining of the acrosomal vesicle (Fig. 4G,H and O,P). These results are in complete agreement with the role of DPL in spermiogenesis 24, 25.

**Figure 5:**
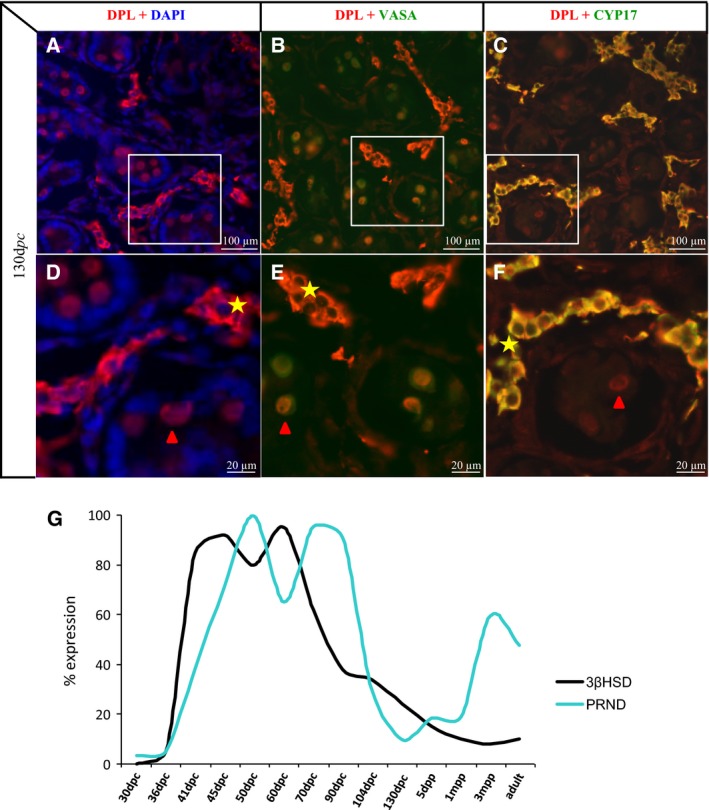
Expression of Leydig cell markers in goat testis. Dpl (A–F), VASA (B, E) and CYP17 (C, F) immunodetections were performed in a goat testis at 130 d*pc*. (D–F) photographs correspond to an enlargement of the white rectangles depicted on (A–C) photographs. (G): 3βHSD (black curve) and *PRND* (blue curve) expression was quantified using real‐time RT‐PCR during testis development in goat and represented on the same graph. At 30 and 36 d*pc* gonads are not dissected from mesonephros. d*pc*, days *post coïtum*; d*pp*, days *post partum*; m*pp*, months *post partum*.

More interestingly, a strong staining is detected in some cells of the interstitial testicular space, specifically in the goat species and at immature stages, as the staining disappears in adult testes (compare Fig. 4I,L with M,N). In order to precisely define this DPL staining in the interstitial testicular compartment, we carried out double IHC with an anti‐CYP17 antibody detecting a Leydig cell‐specific marker corresponding to a key enzyme of steroid synthesis, the cytochrome P_450_ 17alpha‐hydroxylase/17,20‐lyase 38. DPL and CYP17 are found colocalized in the same interstitial cells (Fig. 5A–F). DPL staining in goat testes disappears between the prepubertal 3‐month and the pubertal 7‐month stages (Fig. 4K–N), suggesting that *PRND* is specifically expressed in the foetal Leydig cell population. These cells disappear after birth and are replaced by adult Leydig cells at puberty 42. Finally, the *PRND* expression profile appear hugely similar to that of *3*β*HSD*, another Leydig cell‐specific marker, until 1 month after birth indicating that during testis development the major part of *PRND* expression is Leydig specific (Fig. 5G). This observation could explain the high testicular levels of *PRND* transcripts detected from 41 d*pc* to 1 m*pp* specifically in goat testes (Fig. 1G) and not in the mouse testes of the corresponding stages, from 12.5 d*pc* to 5 d*pp* (Fig. 1F). During the prepubertal period, spermatogenesis starts (25 d*pp* in mice, 3 m*pp* in goats), the number of germ cells increases and more importantly differentiated meiotic germ cells expressing *PRND/Prnd* appear. From these prepubertal stages, the *PRND/Prnd* expression profiles are similar between mice and goats (Fig. 1F,G), but diverge from that of *3*β*HSD* in goats (Fig. 5G). It indicates that around puberty *PRND* expression increases in postmeiotic germ cells and disappears from foetal Leydig cells because of their own disappearance.

In conclusion, we report the differential expression of the three members of the prion protein gene family in the developing gonads of mice and goats. Only relatively low levels of expression were detected for Sho, an observation that might relate with the lack of reproductive‐associated phenotype in mouse *Sprn*‐knockout mice 8, 19. By contrast and in addition to its conserved role in spermiogenesis, *PRND* seems to be a key marker of foetal Leydig cells in goats. This observation adds *PRND* to the list of genes having potentially different biological roles in mice and humans or ruminants (see 39, 43 for recent examples). The present results highlight that *PRND* is a key candidate gene for functional studies in goats because its involvement in foetal Leydig cells cannot be studied in the widely used mouse mammalian model. Deciphering this role may have important implications for human reproduction. In order to determine if *PRND* could be a crucial actor of foetal Leydig cell development, its targeting is currently under way in the goat species by using genome editing technologies, recently proven successful by us in this species 39.

## Author contributions

AAB, MP, KMG, JLV and EP participated in the conception of the study, interpretation of data and in the drafting of the article. BP and JC participated in the design and realization of animal experiments. AAB, ME, MA and FMT performed experiments.
